# Potential probiotic *Lactobacillus delbrueckii* subsp. *lactis* KUMS-Y33 suppresses adipogenesis and promotes osteogenesis in human adipose-derived mesenchymal stem cell

**DOI:** 10.1038/s41598-024-60061-2

**Published:** 2024-04-27

**Authors:** Farjam Goudarzi, Amir Kiani, Yousef Nami, Azin Shahmohammadi, Adel Mohammadalipour, Afshin Karami, Babak Haghshenas

**Affiliations:** 1 Regenerative Medicine Research Center, Kermanshah University of Medical Sciences, Kermanshah, Iran; 2 Department of Food Biotechnology, Branch for Northwest and West Region, Agricultural Biotechnology Research Institute of Iran, Agricultural Research, Education and Extension Organization (AREEO), Tabriz, Iran; 3 Student Research Committee, Kermanshah University of Medical Sciences, Kermanshah, Iran; 4 Department of Clinical Biochemistry, Isfahan Pharmaceutical Sciences Research Center, Isfahan University of Medical Sciences, Isfahan, Iran; 5 Departments of Hematology and Blood Banking, Faculty of Allied Medicine, Iran University of Medical Sciences, Tehran, Iran

**Keywords:** Osteogenesis, Adipogenesis, Human Adipose-derived stem cells, Probiotics, *Lactobacillus*, Biotechnology, Cell biology, Microbiology

## Abstract

Today, probiotics are considered to be living microorganisms whose consumption has a certain number of beneficial effects on the consumer. The present study aimed to investigate the effect of a new probiotic extract (*Lactobacillus delbrueckii* subsp. *lactis* KUMS Y33) on the differentiation process of human adipose-derived stem cells (hADSCs) into adipocytes and osteocytes and, as a result, clarify its role in the prevention and treatment of bone age disease. Several bacteria were isolated from traditional yogurt. They were evaluated to characterize the probiotic’s activity. Then, the isolated hADSCs were treated with the probiotic extract, and then osteogenesis and adipogenesis were induced. To evaluate the differentiation process, oil red O and alizarin red staining, a triglyceride content assay, an alkaline phosphatase (ALP) activity assay, as well as real-time PCR and western blot analysis of osteocyte- and adipocyte-specific genes, were performed. Ultimately, the new strain was sequenced and registered on NBCI. In the probiotic-treated group, the triglyceride content and the gene expression and protein levels of C/EBP-α and PPAR-γ2 (adipocyte-specific markers) were significantly decreased compared to the control group (*P* < *0.05*), indicating an inhibited adipogenesis process. Furthermore, the probiotic extract caused a significant increase in the ALP activity, the expression levels of RUNX2 and osteocalcin, and the protein levels of collagen I and FGF-23 (osteocyte-specific markers) in comparison to the control group (*P* < *0.05*), indicating an enhanced osteogenesis process. According to the results of the present study, the probiotic extract inhibits adipogenesis and significantly increases osteogenesis, suggesting a positive role in the prevention and treatment of osteoporosis and opening a new aspect for future *in-vivo* study.

## Introduction

Bone is a dynamic tissue that provides mechanical protection, support for soft tissues, and enables movement. In addition, it maintains systemic mineral balance and is linked to hematopoiesis due to its close relationship with bone marrow cells^1^. Osteoporosis is a disease that leads to decreased bone mass due to imbalance of osteogenesis and adipogenesis. Proteins such as RUNX2 and PPARγ are key initiators for osteogenesis and adipogenesis, respectively, regulating the molecular pathway and the disease will manifest in accordance with decreased osteogenesis and increased adipogenesis^2^.

Many factors in nutritional diet affect the osteoporosis. Calcium and Vitamin D3^3^ along with dietary lipids^4^ and coenzyme Q10 are the examples of these factors. Many studies in the current scientific literature confirm the beneficial effects of probiotics on the human body. Probiotics are living microorganisms that have health benefits for the host when used in sufficient amounts. The health benefits of these compounds include increasing resistance to gastrointestinal infections^5^, lowering blood lipid concentrations^6^, stimulating the immune system^7^, controlling body weight, controlling obesity, improving energy metabolism, increasing insulin sensitivity, treating obesity, and treating insulin resistance^8^, as well as lowering LDL-cholesterol^9^. Among the probiotics some are more prominent.

*Bifidobacterium* and *Lactobacillus* have shown anti-obesity effects in mouse obesity models^10,11^. *Bifidobacterium animalis* subsp. *lactis* CECT 8145 has a strong fat reduction capacity that regulates fat metabolism in obese mouse models^12^. *B. animalis* subsp. *lactis* I-2494 can reduce body weight and balance intestinal microbial composition^13^. It has been reported that Akkermansia muciniphila^14^ can reduce adipose tissue mass and inhibit obesity^15,16^. The mechanisms by which probiotics improve obesity are associated with fat metabolism, increased insulin sensitivity, and intestinal microbial composition^17^. The growing evidence exhibits that the anti-inflammatory activity of probiotics has a beneficial influence on bone homeostasis^18^. Probiotics in animals inhibit bone loss caused by diabetes, periodontal disease, and estrogen deficiency^19^.

The expansion of marrow adipose tissue (MAT) and the related lipotoxicity are significant causes of bone loss and atrophy of hematopoietic bone marrow (HBM). The MAT is mainly distributed in bones of skeletal system and is different from other adipose tissue of the body, hence it is heterogeneous^20^ MAT-related lipotoxicity damages the osteoblast’s survival and function, directing the osteoblast toward a pro-adipogenic/pro-apoptotic phenotype, leading to osteoporosis. The accumulation of MAT causes a reduction in bone formation in the vicinity of MAT and a decrease in the mechanical strength of bone^21^. Some investigations also evaluate an appropriate perspective to clarify the role of probiotics itself in the postmenopausal osteoporosis in women, suggesting a potential role of probiotics^22^. These probiotics may influence the MAT by secreting some compound into the blood and affecting the bone age diseases such as osteoporosis.

The presence of probiotics in calcium-rich dairy products may explain the osteogenic and anti-adipogenic properties of these foods, which may not be due to calcium alone. Therefore, studying the effect of extracts that are secreted by these bacteria on stem cell differentiation can greatly improve our view of the usefulness of this food and the living organisms within it. The present research aimed to evaluate the effect of the prepared probiotic extract on adipogenesis and osteogenesis of hADSCs by investigation specific molecular markers in both osteogenesis and adipogenesis pathways and, as a result, clarify its role in the prevention and treatment of osteoporosis in a new topic that has been addressed in a small way before^23^.

## Materials and methods

### Sampling and isolation of bacterial strains

Seventy samples of traditional fermented yogurt randomly collected from different parts of Kermanshah province in the west of Iran were transported to the laboratory separately to isolate probiotic bacterial strains and stored in the refrigerator (4 °C). Five grams of each sample were well dissolved in 95 mL of sterile trisodium citrate solution, and then 1 mL of the mentioned solution was added to 20 mL of de Man Rogosa Sharpe (MRS) broth medium. Bacterial strains were propagated under anaerobic growth conditions for 24 h at 37 °C in MRS broth and spread on a MRS agar culture medium. Then, the proliferated bacterial colonies were isolated and subjected to initial biochemical and morphological assessments, including a catalase test, cell morphological analysis, and gram staining, for further investigation^24^.

### Survival in simulated gastrointestinal conditions

To evaluate the tolerance of isolated strains to high bile salt concentrations and low pH, 5 mL of each bacterial culture was grown in MRS medium for 24 h and centrifuged at 3000×*g* for 10 min. Then, the supernatant was discarded, and the settled cells were suspended with gentle stirring for respectively 4 and 3 h in 10 mL of solutions containing high bile salt (0.3% w/v oxgall, pH 6.8, and 37 °C) and low pH (pH 2.5 at 37 °C). By measuring the optical density [1] values of the treated and untreated strains by a spectrophotometer (Eppendorf, Germany) at 600 nm and based on the method described by Yang et al.^25^, the bacterial survival rates were estimated as follows: [OD (after treatment)/OD (before treatment)] × 100%.

To assess survival under gastric conditions, pepsin at a final concentration of 5% (w/v) was added to selected strains with an initial cell concentration of 1.9–3.8 × 10^9^ CFU/mL (pH 2.5 at 37 °C), and the cells were incubated at 110 × g for 2 h. Also, to create intestinal digestive conditions, a solution of bile salts and pancreatin in concentrations of 0.3 and 0.1% (w/v) (pH 6.0 at 37 °C, respectively) was added and incubated for 3 h with gentle stirring at 110×*g*. Before and after treatment, the samples were diluted and cultured in three replicates on MRS agar medium for 48 h at 37 °C, then bacterial clones were counted. The survival rate was calculated using the following equation:

$${\text{Survival}}\;{\text{rate}}\;\left( \% \right) = \left( {{\text{log}}\;{\text{CFU}}\;{\text{N}}_{{1}} /{\text{log}}\;{\text{CFU}}\;{\text{N}}_{0} } \right) \times {1}00\%$$

where N_1_ corresponds to the total clones grown in harsh conditions and N_0_ corresponds to the total counted clones before treatment^24^.

### Probiotic assessment

To measure the ability of cholesterol uptake by probiotic strains, the cells were inoculated for 20 h (37 °C) in the MRS culture medium enriched with water-soluble cholesterol (polyoxy ethanyl cholesteryl sebacate; Sigma (150 μg/mL)) and bile salt (bile oxgall (0.3%)). Then, the bacterial cells were collected by centrifugation at 4000×*g* for 20 min, and the remaining cholesterol in the aqueous phase was measured by the o-phthaldehyde method 18. To check cell surface hydrophobicity, the overnight culture of bacterial cells was centrifuged at 4000 × g for 20 min, and the settled bacteria (10^8^ CFU/mL) were suspended in 5 mL of PBS. The initial absorbance was measured at 600 nm (A_0_). Then, the bacterial suspension was treated with 1 mL of xylene or toluene (Merck, Germany) by vortexing for 2 min. The created phases were separated at 37 °C for 1 h, and the absorption of the aqueous phase was measured (A_1_). Finally, the cell surface hydrophobicity was determined as a percentage through the formula (1 − A_1_/A_0_) × 100^26^.

To measure the adherence ability of probiotics to human epithelial cells, CaCO_2_ cells were planted on glass sheets and placed in 6-well culture plates. After 24 h of incubation at 37 °C (5% CO_2_), the monolayer cells were washed twice with sterile PBS (pH 7.4), and 10 mL of bacterial suspension (1 × 10^7^ CFU/mL) was added to each plate and incubated at 37 °C for 2 h. Then, it was washed three times with PBS buffer (pH 7.4) to remove non-adherent bacteria. Adherent bacteria were detached using trypsin–EDTA solution (0.05%) and re-suspended in 10 mL of saline solution. Then, serial dilutions of bacteria were cultured on MRS agar and incubated at 37 °C for 24 h. The adhesion percentage was determined by comparing the number of attached bacteria to the total number of examined bacterial cells 20.

The auto-aggregation ability was calculated by the Angmo et al.^27^ method and through the equation % = 1 − (A_t_/A_0_) × 100. Also, the co-aggregation against two pathogens was determined based on the Zuo et al.^28^ method and based on the equation % = (A_0_ − A_t_)/A_t_ × 100, where A_0_ indicates absorption at time 0 and at indicates absorption at time t.

### Anti-pathogenic activities

According to the agar well diffusion method, 1.5 10^8^ CFU/mL (half McFarland) of seven common human pathogens, including *Escherichia coli* (PTCC 1276), *Klebsiella pneumoniae* (PTCC 1053), *Listeria monocytogenes* (ATCC 13932), *Yersinia enterocolitica* (ATCC 23715), *Shigella flexneri* (PTCC 1234), *Staphylococcus aureus* (ATCC 25923), and *Bacillus subtilis* (ATCC 19652), were cultured on Mueller–Hinton agar culture medium. The wells were created on the inoculated medium and were incubated with 70 µL of cultivated overnight probiotic filtered supernatant in different pH (neutral and natural pH) and conditions (catalase and proteinase K treated) at 37 °C for one night, then the inhibition zones were measured with a digital caliper^29^.

### Antibiotic susceptibility

In the disc diffusion method, probiotic strains are cultured overnight in the MRS agar culture medium at 37 °C. Then, the discs of 10 widely used and clinically important antibiotics, including vancomycin (30 μg), streptomycin (10 μg), kanamycin (30 μg), tetracycline (30 μg), erythromycin (15 μg), ampicillin (10 μg), clindamycin (2 μg), chloramphenicol (30 μg), cephalexin (30 μg), and gentamycin (10 μg) were placed on the inoculated media, and the diameter of the inhibition zones was measured with a digital caliper^30^.

### Safety characterization

Three classifications were used to evaluate the hemolytic activity of bacterial strains: (1) bacterial colonies without halos were classified as γ-hemolysis; (2) bright halos around the colony were classified as β-hemolysis; and (3) green halos around the colony were classified as α-hemolysis. Meanwhile, multiplex PCR techniques based on the program: initial denaturation at 95 °C for 5 min followed by 35 cycles including denaturation (95 °C for 60 s), annealing (54 and 56 °C for 60 s), extension (72 °C for 60 s), and final extension at 72 °C for 5 min to detect ten potential virulence genes, including esp, *ace, ccf, gel E, cylA, cylM, cylB, agg, cpd*, and *cob*, were used in bacterial strains. Also, in this study, *Enterococcus faecium* (ATCC 8043) and *Enterococcus faecalis* (ATCC 29212) were used as control strains^31^.

### Bacterial identification

The extracted bacterial genomic DNA using primers Hal-6F (5′-AGAGTTTGATCMTGGCTCAG-3) and Hal-R6 (5′-TACCTTGTTAGGACTTCACC-3′) and based on the PCR cycles, which included initial denaturation at 95 °C for 4 min and then 32 cycles including 94 °C for 1 min, 58 °C for 1 min, 72 °C for 95 s, and the final extension at 72 °C for 5 min, was amplified. Then, to identify the bacterial strains, the amplified and purified DNA was sequenced by the Macrogene Company (South Korea), and the results were compared with the deposited sequences stored in the NCBI and GenBank sites^32^.

### The bacterial extract preparation

Bacterial strains were propagated under anaerobic growth conditions for 24 h at 37 °C in MRS broth to reach a cell concentration of 1–10 × 108 CFU/mL. The bacterial extracts were separated by centrifugation at 8000×*g* for 20 min at 4 °C, and their pH was adjusted to 7.2 by adding NaOH. Bacterial supernatants were then carefully filtered through 0.22 µm syringe filter units (Nalgene, Sigma-Aldrich) and prepared for use in cell differentiation assays 27.

### ADSCs isolation

Lipoaspirates are disposed of as waste after liposuction surgery, so they are a great source of MSCs. They were collected after the consultant met with the patient (female through 35–40 years old) to acquire the contest under the supervision of the Ethics Committee of Kermanshah University of Medical Sciences (IR.KUMS.REC.1399.597).

The ADSCs were extracted from lipoaspirates according to the procedure explained by Goudarzi et al.^33^. For the enzymatic digestion, the lipoaspirate was rinsed three times with PBS (pH 7.45) containing 5% penicillin/streptomycin (pen/strep) and then put in a solution of collagenase type I (1 mg/mL) for 60 min. To neutralize the collagenase activity, DMEM‐F12 supplemented with 10% fetal bovine serum (FBS) was used. Next, the centrifugation was carried out at 500×*g* for 6 min, and the obtained cell pellet was cultured until the fourth passage.

### ADSCs immunophenotyping

The fourth passage cells were trypsinized (0.25%-2 mM EDTA) and then washed extensively with PBS (0.1 M, pH 7.32). Three positive markers, including CD73, CD105, and CD29, and two negative markers, including CD34 and CD45, were considered to characterize the MSCs. Cells were directly incubated with fluorophore-conjugated antibodies for one hour at room temperature and then washed three times with PBS. The signal and gating were performed by a flow cytometer (Attune NxT, Thermo Fisher Scientific, USA) against iso-type controls for the antibodies.

### ADSCs multipotency

For confirmation of the ADSCs differentiation capacity, they were differentiated into three lineages: chondrocyte, adipocyte, and osteocyte. The fourth-passage ADSCs at 80–90% confluency were incubated in adipogenic medium (DMEM‐F12, pen/strep (1%), FBS (10%), insulin (10 µg/mL), IBMX (0.5 mM), dexamethasone (1 µM), and indomethacin (200 µM) for 14 days), chondrogenic medium (DMEM‐F12, pen/strep (1%), FBS (5%), ITS (1%), IBMX (0.5 mM), dexamethasone (0.1 µM), 2‐phospho-l-ascorbic acid (50 µg/mL), and TGF‐β3 (10 ng/mL) for 21 days), and osteogenic medium (100 nM dexamethasone, 50 μg/mL ascorbic acid, and 10 mM β-glycerophosphate sodium for 28 days). All media were replaced with fresh ones every 3 days. Tri-lineage differentiation of ADSCs was confirmed using Alcian blue (chondrogenesis), Alizarin red (osteogenesis), and Oil Red O (adipogenesis) staining.

### Probiotic treatment

The Y33 strain was chosen due to our preliminary evaluation (data are not shown) to assess the differentiation trend. ADSCs were seeded at a density of 100,000 cells per well in a 24-well plate. At 90% confluency, the osteogenesis and adipogenesis of ADSCs were induced based on the procedure described earlier. The protein concentration of the probiotic extract was measured via Bradford assay. In experimental groups, 5, 10, and 20 µg/mL of Y33 strain extract protein were added to the differentiation media. Control groups received no probiotic extract.

### Triglyceride content and alkaline phosphatase (ALP) activity assay

The triglyceride content and alkaline phosphatase activity were measured using the Pars Azmoon kits (Pars Azmoon Co., Tehran, Iran) based on the manufacturer’s instructions. After the treatment period, the cells were washed, trypsinized, and lyzed by PBS triton X100 0.5%. Then, the supernatant was collected after centrifugation at 6000×*g* and frozen at 80 °C until the assay day.

### Real-Time PCR

The expression of adipocyte-specific genes such as PPAR-γ2 and C/EBP-α, and osteocyte-specific genes such as RUNX2 and osteocalcin was assessed by the real-time PCR assay. To this end, the total RNAs of differentiated cells were extracted using the Kiazol reagent (Kiazist Life Sciences, Iran) based on the manufacturer’s instructions. The quantity and quality of isolated RNAs were confirmed by the NanoDrop One UV–Vis Spectrophotometer (Thermo Scientific). In the next step, the cDNAs were synthesized using the cDNA synthesis kit (GeneAll, South Korea) according to the manufacturer’s instructions. The forward and reverse primer sequences listed in Table 1 were employed for the real-time amplification of selected genes by the LightCycler® 96 System (Roche, Germany). The 2^−△△Ct^ method was applied to calculate the relative gene expressions (fold changes), and the expression of RNA Polymerase II (a reference gene) was used to normalize the data.

**Table 1:** Primer sequences for adipogenic and osteogenic differentiations.

Gene name	Sequences	T_a_
PPAR-γ_2_	F- CTATTGACCCAGAAAGCGAT R- CGTAATGTGGAGTAGAAATGC	54
C/EBP-α	F- GGTGCGTCTAAGATGAGGGG R- CATTGGAGCGGTGAGTTTGC	55
RUNX2	F- CTCCCCAGGCCAAACACA R- TCCGAGGGCTACCACCTTGA	55
Osteocalcin	F- CACCGAGACACCATGAGAGC R- CTGCTTGGACACAAAGGCTGC	54
RPII	F- GCACCATCAAGAGAGTCCAGT R- ATTTGATGCCACCCTCCGTCA	57

### Western Blotting

Cells from control and treated groups were lysed using radioimmunoprecipitation assay (RIPA) buffer (50 mM Tris–HCl pH 7.2, 1% NP40, 150 mM NaCl, 25 mM MgCl2, 0.1% SDS, 1 mM PMSF, 0.5% DOC) containing a protease inhibitor cocktail (Santa Cruz Biotechnology, Inc., Dallas, USA). The bicinchoninic acid (BCA) assay was used to measure the protein content. Fifty micrograms of protein from each sample were resolved using sodium dodecyl sulfate–polyacrylamide gel electrophoresis (SDS-PAGE) and then transferred to a nitrocellulose membrane. Membranes were rinsed with TBS buffer (20 mM Tris–HCl, 500 mM NaCl, pH 7.4) and blocked with 3% BSA dissolved in TBST buffer (TBS buffer and 0.5% Tween 20) at room temperature for 2 h. Afterward, they were incubated with antibodies against adipocyte markers, including goat polyclonal anti-PPAR-γ2 and anti-C/EBP-α antibodies (1:500 in TBST buffer; Santa Cruz Biotechnology, USA), and antibodies against osteocyte markers, including goat polyclonal anti-Collagen I and anti-FGF-23 antibodies (1:500 in TBST buffer; Santa Cruz Biotechnology, USA), overnight at 4 °C. Also, β-actin was employed as an endogenous control. In the next step, membranes were rinsed thrice with 0.5% TBST buffer and incubated with an HRP-conjugated anti-goat secondary antibody (1:10,000 in TBST buffer; Santa Cruz Biotechnology, USA) for 1 h. The immunoreactive bands were visualized using an enzyme-linked chemiluminescence detection kit (Kiazist Life Sciences, Iran).

### Statistical analyses

The experiments were performed three times independently and based on a completely randomized design with three replications for each group, and the data were analyzed by SPSS Statistics 19 software using ANOVA and ad-hoc Tukey tests. Meanwhile, the *P* < *0.05* level was considered significant for the means of the data. The normality of the data was assessed with Shapiro–wilk test and due to a normal distribution ANOVA was chosen. Homogeneity of variance was also assessed so ad-hoc Tukey test was also considered in accordance to compare means of each group.

## Result and discussion

### Morphological and biochemical assessment

The isolation of lactic acid bacteria represents a pivotal stage in probiotic research. It facilitates meticulous investigation, precise characterization, and well-informed scientific deductions. This step is foundational in unraveling the probiotic community and accomplishing the research goals of this study. Recent research endeavors utilize this process to acquire knowledge about probiotic diversity, health implications, and practical applications^34^.

After anaerobic growth in the specific culture medium (MRS), 54 hemispherical white to cream-colored bacterial colonies, were isolated. Among them, 30 colonies which included catalase-negative and gram-positive rod-shaped or spherical bacteria, were considered LAB bacteria and selected for further analysis.

The selection criteria for LAB are multifaceted and crucial to guarantee their effectiveness and safety. The process of choosing LAB strains for this study entails a thorough assessment of their characteristics, functionality, and safety profile. These criteria collectively ensure that these probiotic strains significantly enhance human health and overall well-being^14^.

### Survival in simulated gastrointestinal conditions

One of the essential criteria for evaluating oral probiotics is their high survivability against the defense systems of the digestive tract, including low pH and bile salts. Due to the similar growth conditions during in vitro and in vivo experiments, the preliminary tolerance assessment of bacteria in the digestive tract can be determined by an in vitro optical density study with the same pH (2.5 for 3 h) and oxgall concentration [0.3% (w/w) for 4 h^35^. The process of selecting probiotic strains based on their tolerance in in vitro simulated gastrointestinal conditions relies on the specific criteria such as low pH and high bile salts conditions^36^. These criteria serve to evaluate the suitability of probiotic candidates within this study for survival and functionality within the digestive tract. Laboratory experiments replicate the challenging conditions of the gastrointestinal tract, and strains that withstand these conditions are more likely to thrive in vivo. Consequently, probiotic strains chosen for their tolerance in simulated gastrointestinal conditions should demonstrate safety, survival capability, and functional attributes relevant to gut health^37^.

Based on the initial assessment of tolerance at harsh conditions related to 30 isolated strains (Table 2) and due to stress adaptation mechanisms, bile salts usually have less lethal effects than acid on bacterial cells, so bacterial strains in the presence of bile salts mostly showed higher tolerance, which was 1–21% more than their tolerance at low pH^38^. There was a large difference in the survival rates in the mentioned conditions, and only 5 strains, including Y12, Y17, Y23, Y33, and Y41, had a survival percentage of more than 91% and therefore were selected for the additional tolerance study (Table 2).

**Table 2:** The survival rates (%) of isolated LAB after 3 h of incubation at pH 2.5 and 4 h of incubation at 0.3% bile salt.

Survival rates (%): ([OD_600_ (3–4 h)/OD_600_ (0 h)] × 100)					
Isolates	Survival rates (%) at pH 2.5	Survival rates (%) at 0.3% bile salt	Isolates	Survival rates (%) at pH 2.5	Survival rates (%) at 0.3% bile salt
Y2	18.06 ± 1.54^i^	38.25 ± 1.0^gh^	Y33	92.57 ± 1.91^a^	93.76 ± 1.68^a^
Y5	21.28 ± 0.96^ h^	42.06 ± 1.69^de^	Y35	37.32 ± 0.95^d^	42.47 ± 1.16^cde^
Y9	24.29 ± 1.33^ g^	39.37 ± 0.94^ fg^	Y39	35.42 ± 2.17^d^	36.48 ± 1.0^hi^
Y12	91.03 ± 2.3^a^	94.81 ± 1.76^a^	Y40	18.50 ± 0.51^i^	20.06 ± 0.64^k^
Y13	37.17 ± 1.74^d^	42.25 ± 0.73^de^	Y41	93.93 ± 1.38^a^	94.29 ± 1.99^a^
Y16	33.41 ± 2.02^e^	35.11 ± 1.2^i^	Y44	31.47 ± 1.03f.	35.33 ± 1.17^i^
Y17	92.17 ± 1.82^a^	93.84 ± 0.25^a^	Y48	38.20 ± 1.03^d^	44.36 ± 0.78^c^
Y19	37.00 ± 1.88^d^	40.64 ± 0.63^ef^	Y53	37.04 ± 1.36^d^	39.87 ± 0.34^fg^
Y21	36.32 ± 0.81^d^	39.86 ± 2.02^fg^	Y57	30.51 ± 1.48^f^	32.39 ± 1.03^j^
Y22	37.37 ± 1.87^d^	42.44 ± 1.11^cde^	Y60	42.53 ± 1.27^c^	43.42 ± 1.33^cd^
Y23	91.73 ± 1.55^a^	93.36 ± 0.83^a^	Y62	33.00 ± 2.44^ef^	35.38 ± 1.15^i^
Y25	37.51 ± 1.49^d^	40.71 ± 0.61^ef^	Y63	22.26 ± 1.90^gh^	32.27 ± 0.92^j^
Y28	30.79 ± 1.6^f^	36.38 ± 0.8^hi^	Y67	31.20 ± 1.63^f^	34.49 ± 1.0^i^
Y29	22.52 ± 1.79^gh^	40.71 ± 1.15^ef^	Y69	30.57 ± 1.37^f^	35.32 ± 1.84^i^
Y30	33.40 ± 2.16^e^	36.62 ± 0.6^hi^	Y70	36.43 ± 1.81^d^	40.85 ± 0.56^ef^

Values shown are means ± standard deviations (n = 3).

*Values followed by the same letters are not significantly different (*P* < *0.05*). Statistical analysis of each formulation was done separately.

All five bacterial strains survived effectively after exposure to simulated digestive conditions (Table 3). Based on the results, these strains had 16–17% higher survival in the digestive conditions of the intestine than in the digestive conditions of the stomach. Meanwhile, the highest survivability in simulated digestive conditions belonged to Y17 and Y33, with survival rates of 83% and 81% in intestinal digestive conditions and 76% and 75% in gastric digestive conditions, respectively (Table 3). Similar to our results, high survival rates in digestive conditions due to the two-layer membrane structure of LAB have been reported for probiotic strains such as *L. lactis* subsp. *cremoris* 44L, *L. plantarum* 15HN, E. faecalis 13C, *E. durans* 39C, and *E. mundtii* 50H 18,31,32. Based on the results, all five bacterial strains maintained their survival after exposure to simulated digestive conditions. As a result, these strains (Y12, Y17, Y23, Y33, and Y41) were selected for further analysis.

**Table 3:** Survival rates (%) under gastric and intestinal digestive conditions, cholesterol uptake, surface hydrophobicity, adherence ability, auto-aggregation (%), co-aggregation (%), and hemolytic activity of isolated LAB.

Isolates	Survival (%) in gastric conditions	Survival (%) in intestinal conditions	Cholesterol uptake	Surface hydrophobicity		Adherence ability	Auto-aggregation (%)	Co-aggregation (%)		Hemolytic activity
				Toluene	Xylene			*S. aureus*	*B. cereus*	
Y12	62 ± 0.86^c^	68 ± 0.29^c^	27.07 ± 1.72^c^	62.23 ± 3.23^ab^	63.37 ± 2.04^b^	4.03 ± 0.49^e^	48.80 ± 1.55^c^	38.70 ± 1.55^c^	37.30 ± 2.61^b^	γ-hemolytic
Y17	76 ± 0.46^a^	83 ± 0.96^a^	25.10 ± 1.55^c^	60.40 ± 2.12^b^	62.50 ± 2.94^b^	26.40 ± 0.65^b^	53.10 ± 1.88^b^	46.50 ± 1.88^b^	34.70 ± 1.39^b^	γ-hemolytic
Y23	68 ± 0.54^b^	75 ± 1.23^b^	30.10 ± 1.47^b^	55.00 ± 2.53^c^	56.60 ± 1.14^c^	12.43 ± 0.50^d^	43.60 ± 2.12^d^	24.30 ± 2.20^d^	13.80 ± 1.96^c^	γ-hemolytic
Y33	75 ± 1.07^a^	81 ± 0.33^a^	63.40 ± 0.73^a^	67.10 ± 1.47^a^	73.23 ± 2.82^a^	48.50 ± 0.57^a^	70.90 ± 2.29^a^	62.10 ± 1.96^a^	61.10 ± 2.12^a^	γ-hemolytic
Y41	60 ± 0.37^c^	67 ± 0.74^c^	19.20 ± 0.90^c^	51.40 ± 1.80^c^	53.30 ± 2.20^c^	18.63 ± 0.45^c^	38.70 ± 1.55^e^	18.20 ± 1.31^e^	9.20 ± 1.71^d^	γ-hemolytic

Values shown are means ± standard deviations (n = 3).

*Values followed by the same letters are not significantly different (*P* < *0.05*). Statistical analysis of each formulation was done separately.

In vitro experiments assessing the survival of probiotic LAB under simulated gastrointestinal conditions are pivotal for evaluating their tolerance within the digestive tract^39^. These laboratory-based studies effectively bridge the gap to real-life digestive processes. While in vitro experiments provide a rapid and ethical means to study probiotic interactions, a combination of in vitro and in vivo studies is indispensable for comprehending probiotic mechanisms in real-world digestive contexts.

### Probiotic assessment

The ability to uptake cholesterol is another important and necessary feature for the introduction and selection of probiotics. Evidence shows that the hypocholesterolemic effects of probiotics can be due to the absorption of cholesterol or the binding of cholesterol to the surface of bacterial cells^40^. The cholesterol-lowering effects of probiotic strains are closely associated with their ability to assimilate cholesterol, as substantiated by existing scientific research^41^. In vivo studies consistently reveal that the consumption of probiotic bacteria, particularly health-promoting LAB strains, results in decreased cholesterol and blood lipid levels. These beneficial effects significantly contribute to overall cardiovascular well-being^42^. Notably, *L. rhamnosus* BFE5264 and *L. plantarum* NR74, both considered potential probiotic strains, exhibit the capacity to inhibit cholesterol uptake and enhance cholesterol efflux within intestinal cells. Consequently, probiotic LAB strains play a pivotal role in managing cholesterol levels, aligning with the objective of promoting cardiovascular health and mitigating the risks associated with elevated cholesterol^43^. The efficiency of cholesterol removal by the probiotic strains from the culture medium (cholesterol absorption) is presented in Table 3. The Y33 strain could uptake 63% of environmental cholesterol (94.5 out of 150 μg/mL) after 20 h of incubation, while the cholesterol absorption of the Y23, Y12, Y17, and Y41 strains was 30, 27, 25, and 19, respectively. The most significant mechanisms of cholesterol reduction by probiotics include the conversion of cholesterol to coprostanol by the reductase enzyme, the incorporation of cholesterol in the cell wall, and the disruption of cholesterol micelle formation in the intestine by de-conjugated bile salts^44^.

Examining the adhesion capacity of probiotics to the hydrophobic phase of the used solvent (surface hydrophobicity) is necessary to determine the ability of bacteria to adhere to the intestinal mucosa, which prevents pathogens from sticking to the intestine and contaminating the digestive system^45,46^. Based on the results, all five bacterial strains showed cell surface hydrophobicity higher than 51%. Meanwhile, the Y33 strain had surface hydrophobicity values of 73.23% (Xylene) and 67.10% (Toluene), which were significantly (*P* < *0.05*) higher than other tested strains (Table 3). Similar to our results, previous studies have shown that probiotic bacteria mainly exhibit acceptable cell surface hydrophobicity, and these studies have also proven the relationship between cell surface hydrophobicity and bacterial adhesion ability^47^. On the other hand, the difference in the production of surface proteins is the main factor in creating a wide range of cell surface hydrophobicity in LAB bacteria^48^. Hydrophobicity is a crucial feature of probiotic bacteria that enhances their clinical significance. This adhesive capability serves as an initial assessment of the probiotic bacteria's ability to engage with epithelial cells^49^. Through adherence and colonization of tissues, probiotic microorganisms can hinder pathogen entry via steric interactions or targeted blockade of cell receptors. The In vitro characteristics of probiotic bacteria, including adhesion and hydrophobicity, may vary based on the host they were derived from. Consequently, the impact of probiotics on host immune cells can be influenced by underlying medical conditions like atopic dermatitis or milk hypersensitivity^50^.

The binding potential of probiotics to intestinal epithelial cells is another selective characteristic of probiotics. Therefore, adherence ability is considered a critical criterion for selecting potential probiotic bacteria^51^. According to the results, strains Y33 (48.50%) and Y17 (26.40%) showed the strongest adhesion to human intestinal CaCO2 cells. Meanwhile, three bacterial strains, Y41 (18.63%), Y23 (12.43%), and Y12 (4.03%), had weak adherence abilities (Table 3). Probiotics, to colonize the gut, must adhere to the intestinal mucosa and not be easily removed from the gut with smoky bowel movements. Our results are consistent with other studies that showed that only some LAB strains, such as Y33 and Y17, could adhere well to Caco-2 cells^52^.

Auto and co-aggregation are two important properties of probiotic bacteria, which are defined as the aggregation of similar or different bacterial species, respectively^38^. These two characteristics are essential for probiotics, as auto-aggregation appears to be associated with the adhesion of probiotics to intestinal epithelial cells, while co-aggregation is a defensive barrier to the establishment and stabilization of pathogenic microorganisms^53^. The auto-aggregation rate of the strains varied from 38.70% to 70.90%. The highest auto-aggregation was obtained for the Y33 and Y17 strains, with a rate of 70.90% and 53.10%, respectively. In addition, strains Y12, Y23, and Y41 showed the lowest rate of cell auto-aggregation, with a rate of less than 49% (Table 3). According to Table 3, the Y33 strain in the presence of *S. aureus* (62.10%) and *B. cereus* (61.10%) had the best co-aggregation ability, which was significantly higher (*P* < *0.05*) compared to other strains. The results confirm this hypothesis: the Y33 and Y17 strains, which had the best hydrophobicity and adhesion to intestinal epithelial cells, also showed the best auto- and co-aggregation abilities.

### Anti-pathogenic activities

One of the expected characteristics of probiotic bacteria is that they have an acceptable inhibitory function against pathogenic agents. Therefore, the anti-pathogenic properties of the selected strains against Gram-positive and Gram-negative pathogens were evaluated. Antagonistic activities of five isolated strains against seven pathogens in different pH (neutral and natural pH) and conditions (catalase and proteinase K treated) are shown in Table 4. According to the results, the Y33 strain displayed significant anti-pathogenic activity and inhibited the growth of all pathogens (Table 4). Meanwhile, Y12, Y17, Y23, and Y41 showed moderate antagonistic activity, and each of them inhibited the growth of three pathogens, including *L. monocytogenes, S. aureus*, and *B. subtilis* (Table 4).

**Table 4:** The inhibitory effect of isolated LAB strains against pathogens. Values shown are means ± standard deviations (n = 3).

Isolates	Conditions	Indicator pathogens Diameter of inhibition zone (mm)						
		*E. coli*	*K. pneumoniae*	*L. monocytogenes*	*Y. enterocolitica*	*S. flexneri*	*S. aureus*	*B. subtilis*
Y12	Neutral pH (6.8)	0	0	9.63 ± 0.21	0	0	12.9 ± 0.8	12.7 ± 0.46
	Acidic pH (5.2)	0	0	12.97 ± 0.61	0	0	16.0 ± 0.66	14.7 ± 0.6
	Catalase treated	0	0	0	0	0	10.8 ± 0.75	0
	Proteinase K treated	0	0	10.43 ± 0.57	0	0	10.97 ± 0.70	11.30 ± 0.98
Y17	Neutral pH (6.8)	0	0	13.67 ± 0.55	0	0	0	17.7 ± 0.38
	Acidic pH (4.8)	0	0	17.27 ± 0.71	0	0	9.1 ± 0.6	21.6 ± 0.66
	Catalase treated	0	0	9.13 ± 0.65	0	0	6.53 ± 0.38	6.17 ± 0.55
	Proteinase K treated	0	0	12.73 ± 0.65	0	0	6.33 ± 0.80	16.1 ± 0.89
Y23	Neutral pH 6.8	0	0	0	0	0	13.13 ± 0.93	0
	Acidic pH (5.1)	0	0	9.13 ± 0.75	0	0	19.77 ± 0.70	10.8 ± 0.36
	Catalase treated	0	0	6.97 ± 0.50	0	0	0	8.37 ± 0.75
	Proteinase K treated	0	0	7.43 ± 0.42	0	0	15.2 ± 0.66	9.77 ± 0.65
Y33	Neutral pH (6.8)	17.27 ± 0.35	0	22.2 ± 0.46	0	15.67 ± 0.45	28.13 ± 0.55	28.17 ± 0.51
	Acidic pH (4.6)	28.33 ± 1.12	18.47 ± 0.74	27.57 ± 0.61	22.33 ± 1.0	23.07 ± 0.55	33.33 ± 0.93	23.33 ± 0.86
	Catalase treated	0	17.23 ± 0.50	27.2 ± 0.26	19.17 ± 0.50	0	27.5 ± 0.60	25.53 ± 0.51
	Proteinase K treated	27.3 ± 0.95	16.97 ± 0.38	0	18.70 ± 0.40	19.03 ± 0.31	0	0
Y41	Neutral pH (6.8)	0	0	5.70 ± 0.66	0	0	9.40 ± 0.30	6.73 ± 0.35
	Acidic pH (3.8)	0	0	11.03 ± 0.61	0	0	12.37 ± 0.45	11.2 ± 0.66
	Catalase treated	0	0	8.00 ± 0.56	0	0	10.37 ± 0.38	8.60 ± 0.62
	Proteinase K treated	0	0	9.33 ± 0.40	0	0	0	8.40 ± 0.10

*Values are mean ± standard error of triplicates. S (strong *r* ≥ 20 mm), M (moderate *r* < 20 mm and > 10 mm), and W (weak ≤ 10 mm).

Probiotics play a crucial role in restraining the growth of pathogens through various mechanisms. They engage in nutrient and adhesion site competition with pathogenic bacteria in the gut, hindering the establishment of harmful pathogens^54^. Additionally, probiotics can neutralize toxins and metabolites produced by pathogenic bacteria, reducing their harmful effects on the host. Certain probiotic strains actively produce bacteriocins, which directly inhibit the proliferation of pathogenic microbes.

Moreover, probiotics enhance the host's immune response, contributing to overall gut health and preventing infections. By strengthening the intestinal barrier, probiotics increase its resilience against pathogen invasion. They can also modify pathogenic toxins and receptors, reducing their impact on the host^55^. Furthermore, probiotics demonstrate enhanced adhesion to the intestinal mucosa, effectively preventing pathogen attachment. Their production of antibacterial compounds further limits the growth of pathogenic organisms.

Overall, probiotics help maintain a balanced microbial community, preventing the overgrowth of pathogens, and stimulate both nonspecific and specific immune responses, aiding in defense against infections^56^. Ultimately, probiotics serve as valuable allies in safeguarding gut health, countering pathogenic invaders, and promoting overall well-being.

Probiotic bacteria belonging to the LAB group prevent the growth and multiplication of pathogens through a combination of different mechanisms such as the production and secretion of hydrogen peroxide (H2O2), organic acids (lactic acid), and inhibitory proteins (bacteriocin)^57^. To find out the type of inhibitory mechanism, the anti-pathogenic activity of the bacterial extracts was evaluated at a natural and neutral pH as well as after being treated with catalase and protease enzymes.

After adjusting the pH to 6.8, the Y33 strain did not show any anti-pathogenic activity against *K. pneumoniae* and *Y. enterocolitica*. Also, strain Y17 was not able to prevent the growth of *S. aureus*, and strain Y23 was not capable of inhibiting *L. monocytogenes* and *B. subtilis*. Therefore, it can be concluded that the inhibition mechanism of these strains against the aforementioned pathogens is due to acid production. On the other hand, the Y33 strain against *S. flexneri* and *E. coli*, the Y12 strain against *L. monocytogenes* and *B. subtilis*, and the Y23 strain against *S. aureus* did not display antagonistic activity after treatment with catalase enzyme. Therefore, it is concluded that the inhibitory nature of these strains against these pathogens is due to hydrogen peroxide production. Finally, after treatment with the proteinase K enzyme and investigation of anti-pathogenic properties, the inhibitory halos for the Y33 strain against *L. monocytogenes, S. aureus*, and *B. subtilis*, as well as the Y41 strain against *S. aureus*, were not observed, which proved the bacteriocin (proteinaceous) nature of the bacterial extracts against the mentioned pathogens (Table 4).

Similar to other studies, this research showed that the anti-pathogenic activities of the LAB strains are mainly related to their ability to produce acid and secrete hydrogen peroxide^58^. Meanwhile, the antagonistic property due to bacteriocin secretion, the same as our results, is observed only against a limited number of Gram-positive and Gram-negative pathogens^59^. These results are in contrast with studies that show that LAB bacteria are only effective on Gram-positive pathogens and have no efficient effect on Gram-negative pathogens due to their outer membrane^60,61^. Some Gram-negative pathogens, such as *Y. enterocolitica* and *K. pneumoniae*, in addition to their high prevalence, show high resistance to antibiotics. Therefore, LAB strains isolated from dairy sources similar to this study can be used against Gram-negative antibiotic-resistant pathogens^62,63^.

### Antibiotic susceptibility

Excessive use of antibiotics by patients has led to the appearance of antibiotic resistance genes in the probiotic intestinal flora, and by transferring these genes to other microorganisms living in the digestive tract, especially pathogens, acute problems related to antibiotic resistance in society are created. Therefore, susceptibility to antibiotics is considered one of the basic characteristics of choosing probiotics^57^.

The results of the antibiotic susceptibility test against 10 clinically important and widely used antibiotics are presented in Table 5. Based on the results, all five strains were susceptible to streptomycin. Also, no strain was susceptible or semi-susceptible to vancomycin, and all strains were resistant to this antibiotic. On the other hand, the best results were observed in Y33 and Y17 strains, which were susceptible or semi-susceptible to 9 (except vancomycin) and 8 (except vancomycin and tetracycline) antibiotics, respectively (Table 5).

**Table 5:** Antibiotic susceptibility profiles of isolated LAB.

Isolated strains	Antibiotics susceptibility zone of inhibition (mm)									
	V	S	K	TE	E	AM	CC	C	CN	GM
Y12	0.00 ± 0.00^c^	18.00 ± 0.71^d^	0.00 ± 0.00^e^	16.67 ± 0.41^c^	14.67 ± 0.82^c^	11.67 ± 1.02^d^	14.00 ± 0.51^b^	17.00 ± 0.64^c^	0.00 ± 0.00^e^	08.67 ± 1.06^c^
Y17	6.33 ± 0.21^b^	20.00 ± 1.02^bc^	16.33 ± 0.32^b^	0.00 ± 0.00^d^	18.33 ± 0.64^b^	16.67 ± 0.94^c^	22.00 ± 0.61^a^	16.33 ± 0.75^c^	14.33 ± 0.52c	10.67 ± 1.25^b^
Y23	0.00 ± 0.00^c^	19.00 ± 0.61^bc^	14.00 ± 0.41^c^	20.67 ± 0.64^b^	0.00 ± 0.00^e^	24.00 ± 1.23^b^	0.00 ± 0.00^c^	20.00 ± 0.87^b^	16.33 ± 0.42^b^	06.33 ± 0.31^d^
Y33	11.67 ± 0.32^a^	24.33 ± 0.52^a^	21.00 ± 0.43^a^	28.00 ± 0.72^a^	25.00 ± 1.05^a^	28.00 ± 1.61^a^	22.00 ± 0.74^a^	27.67 ± 0.98^a^	18.67 ± 0.34^a^	12.33 ± 0.24^a^
Y41	0.00 ± 0.00^c^	20.67 ± 0.42^b^	11.67 ± 0.24^d^	0.00 ± 0.00^d^	13.67 ± 1.04^d^	0.00 ± 0.00^e^	14.33 ± 1.02^b^	0.00 ± 0.00^d^	11.67 ± 0.73^d^	0.00 ± 0.00^e^

Erythromycin results based on R ≤ 13 mm; I: 13–23 mm; S ≥ 23 mm.

Gentamycin results based on R ≤ 6 mm; I: 7–9 mm; S ≥ 10 mm.

Vancomycin results based on R ≤ 12 mm; I: 12–13 mm; S ≥ 13 mm.

I: intermediate (zone diameter, 12.5–17.4 mm); R: resistant (zone diameter, ≤ 12.4 mm); S: susceptible (zone diameter, ≥ 17.5).

*V* vancomycin, *S* streptomycin, *K* kanamycin, *TE* tetracycline, *E* erythromycin, *AM* ampicillin, *CC* clindamycin, *C* chloramphenicol, *CN* cephalexin, *GM* gentamycin.

*Values are mean ± standard error of triplicates.

^a–e^Means in the same row with different lowercase letters differed significantly (*P* < *0.05*).

High susceptibility to antibiotics in five isolated strains (Y12, Y17, Y23, Y33, and Y41) is probably due to the limited use of animal antibiotics in the rural areas of Kermanshah province where the samples (yogurt) were collected. But contrary to these results, high antibiotic resistance in LAB bacteria isolated from traditional dairy products has been reported by other researchers^58–60^. All five isolated strains showed resistance to vancomycin. On the other hand, low resistance to other antibiotics was observed in the strains (Table 5). Various probiotic strains in the LAB group, such as the *Lactobacillus* genus, mainly carry vancomycin resistance genes, which confirm our results^61,62^.

Scientific evidence suggests that antibiotic resistance genes found in probiotic bacteria have the potential to transfer to other commensal and pathogenic bacteria. Recent investigations have uncovered instances where certain probiotic strains display resistance to multiple antibiotics, which differs from our own findings^64^. A comprehensive study examining the antibiotic susceptibility of commercially available probiotic food supplements identified strains resistant to penicillin G, ampicillin, and ceftazidime. It is recommended that probiotic companies thoroughly evaluate antibiotic susceptibility across a wide range of antibiotics when selecting microorganisms for probiotic use^65^. Additionally, analyzing the complete genome sequence can help predict antibiotic resistance genes.

Another study focused on evaluating potential probiotic LAB strains, revealing varying resistance patterns across different antibiotic classes based on both phenotypic and genotypic profiles. While the issue of antibiotic resistance in probiotics requires attention, it may not necessarily pose a safety risk if mutations or intrinsic resistance mechanisms account for the observed resistance^66^. However, the presence of specific antibiotic resistance determinants on mobile genetic elements does raise significant safety concerns. Therefore, it is essential for probiotic manufacturers to carefully consider antibiotic resistance profiles and potential safety implications when developing and selecting probiotic strains for commercial use^67^.

### Safety characterization

Hemolytic activity, a biological process in which certain organisms possess the ability to lyse or break down red blood cells, is a critical factor to consider when studying probiotics. The absence of such activity in probiotics underscores their safety, indicating their non-pathogenic nature^68^. This characteristic is a fundamental criterion in evaluating and selecting probiotics for their potential health benefits. Specifically, these strains lack the ability to induce disease by harming the host's red blood cells. The absence of hemolysis, which triggers the breakdown of red blood cells, ensures that probiotic strains do not exhibit virulence. This reassures consumers and healthcare providers about the safety and reliability of probiotic products for promoting health and well-being^69^. The probiotics did not create a clear halo on the blood agar culture medium, indicating that the bacterial strains had no hemolytic activity and were classified as γ-hemolytic (Table 3). The tested strains did not have α or β-hemolytic properties and only showed γ-hemolytic activity. Other research has shown that most LAB strains, similar to our results, have no hemolytic activity. But according to the guidelines, the lack of blood cell hemolysis does not prove probiotic strains' safety^70,71^.

The presence of virulence genes encoding ten known disease-causing factors was investigated in the probiotic strains. One of the bacterial toxins expressed by some LAB strains is cytolysin. Therefore, the absence of the cytolysin-coding gene is a desirable feature for probiotics used in the food industry. In this study, the bacterial strains did not carry cytolysin-coding virulence genes, including *cylA* and *cylB*. PCR amplification results showed that the Y33 and Y17 strains did not contain any of the mentioned virulence genes. While the Y12 strain showed the presence of the *esp* gene and the Y23 strain had the *cpd* gene. Meanwhile, the Y41 strain carried *ccf*, *esp*, and *cpd* virulence genes. These results follow the results of other research, which generally shows that the presence of virulence genes in LAB strains is lower than in other bacterial strains^63,72^. Cytolysins are a class of toxins produced by specific bacteria, capable of causing damage to host cells by creating pores in their membranes. Our research findings confirm the absence of genes encoding for cytolysin in probiotics. This absence ensures that these beneficial microorganisms do not produce harmful toxins that could potentially harm host cells. This is crucial because cytolysin-induced damage can lead to cell death and contribute to the development of diseases^73^. Moreover, the lack of these genes reduces the risk associated with the horizontal transfer of virulence factors to other bacteria within the gut microbiota. This absence enhances the overall safety and effectiveness of probiotics. By eliminating the potential for toxin production and the transfer of harmful genes, probiotics can offer a safer and more reliable means of supporting gut health and overall well-being^74^.

### Bacterial identification

According to FAO/WHO guidelines, the analysis and identification of probiotic bacteria using 16S-rRNA gene amplification and sequencing can be considered an accessible, cost-effective, and suitable technique compared to other costly and time-consuming molecular techniques^45^.

Amplified PCR fragments belonging to the 16S-rRNA gene of the probiotic strains that showed the best results were sequenced. Based on the blasting of the sequences, the Y12 strain belongs to *Lactiplantibacillus plantarum* (accession number OQ826785), the Y17 strain belongs to *Lactobacillus acidophilus* (accession number OQ826808), the Y23 strain belongs to *L. plantarum* (accession number OQ826809), the Y33 strain belongs to *Lactobacillus delbrueckii* subsp. *lactis* (accession number OQ826810), and strain Y41 belonged to *Lacticaseibacillus casei* (accession number OQ826827). Therefore, based on the results, all five probiotic strains isolated from traditional fermented yogurt were well analyzed and identified up to the strain level by 16S-rRNA gene sequencing. Similar to our results, 16S-rRNA gene sequencing has effectively been used to identify LAB strains belonging to the *Lactobacillus* genus isolated from fermented dairy products^46^. The utilization of the 16S-rRNA gene sequencing method holds significant importance in the realm of probiotic applications. This method allows for the precise identification of bacterial species and strains, which is crucial in probiotics due to the strain-specific health benefits they offer. By identifying these species and strains, it becomes possible to assess probiotic safety effectively, thereby avoiding the inclusion of known pathogenic strains in formulations^29^.

Furthermore, the specific health benefits associated with identified species and strains can inform targeted probiotic formulation, enhancing their efficacy. The 16S-rRNA gene sequencing method also plays a vital role in quality control during probiotic production. It ensures the inclusion of correct strains and helps in preventing contamination. Therefore, the use of 16S-rRNA gene sequencing for species and strains identification, as demonstrated in our results, has profound implications for probiotic safety, efficacy, quality control, and the prediction of functional potential^32^.

### ADSCs characterization

**Figure 1:**
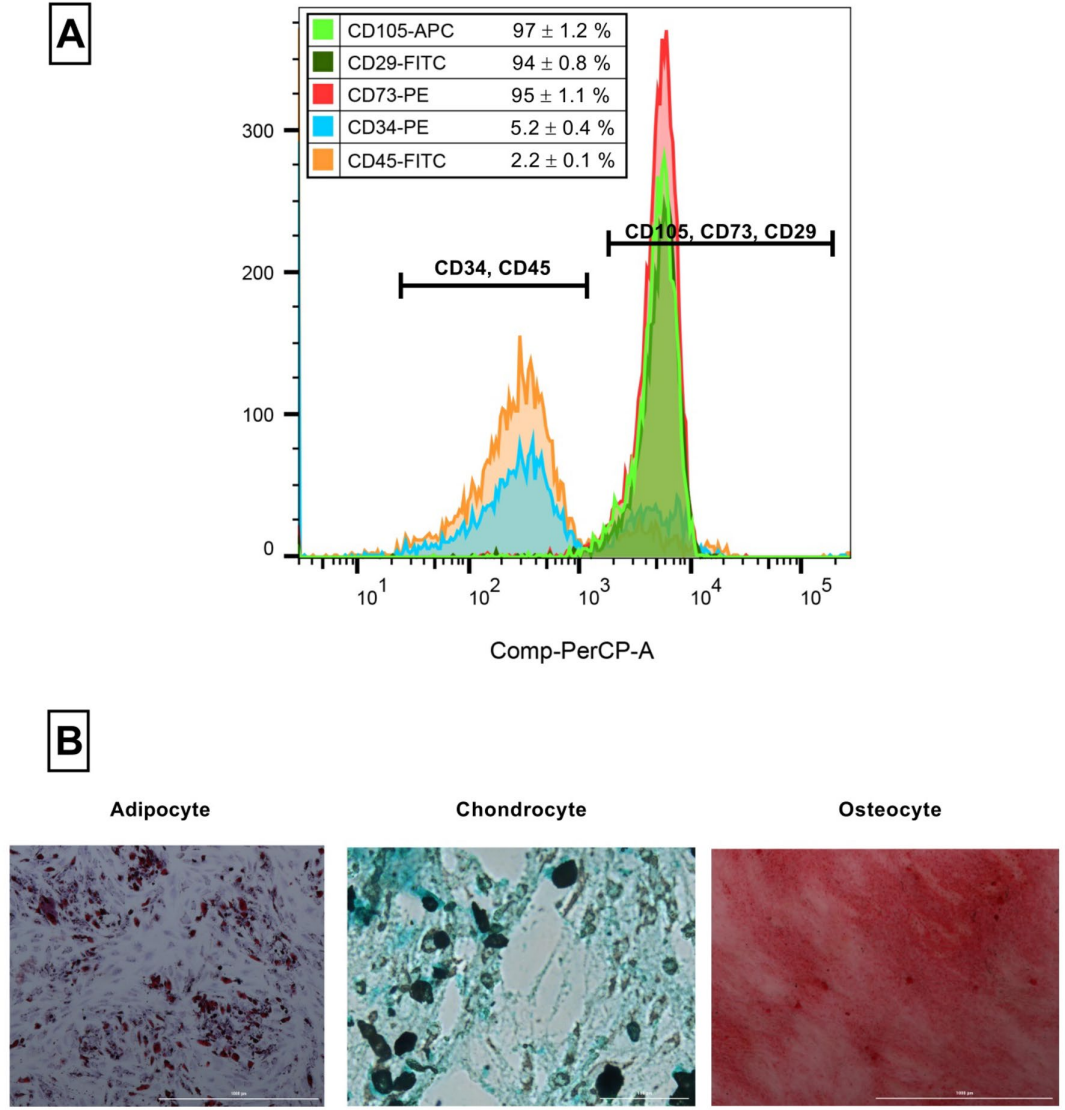
(**A**) Flow cytometric characterization of ADSCs. (**B**) Tri-lineage differentiation of the ADSCs. The white bars under microscopy images are equal to 1000 µm.

Flow cytometry analysis (Fig. 1A) demonstrated a high expression of MSC positive markers, including CD105 (97 ± 1.2%), CD73 (95 ± 1.1%), and CD29 (94 ± 0.8%), and a low expression of negative markers, including CD45 (2.2 ± 0.1%) and CD34 (5.2 ± 0.4%). Alcian blue, alizarin red, and oil red O staining results indicated the successful differentiation of ADSCs into chondrocytes, osteocytes, and adipocytes (Fig. 1B). So, the isolated cells are phenotypically the AD-MSCs according to ISCT^75^.

### Osteogenesis and the extract treatment

**Figure 2:**
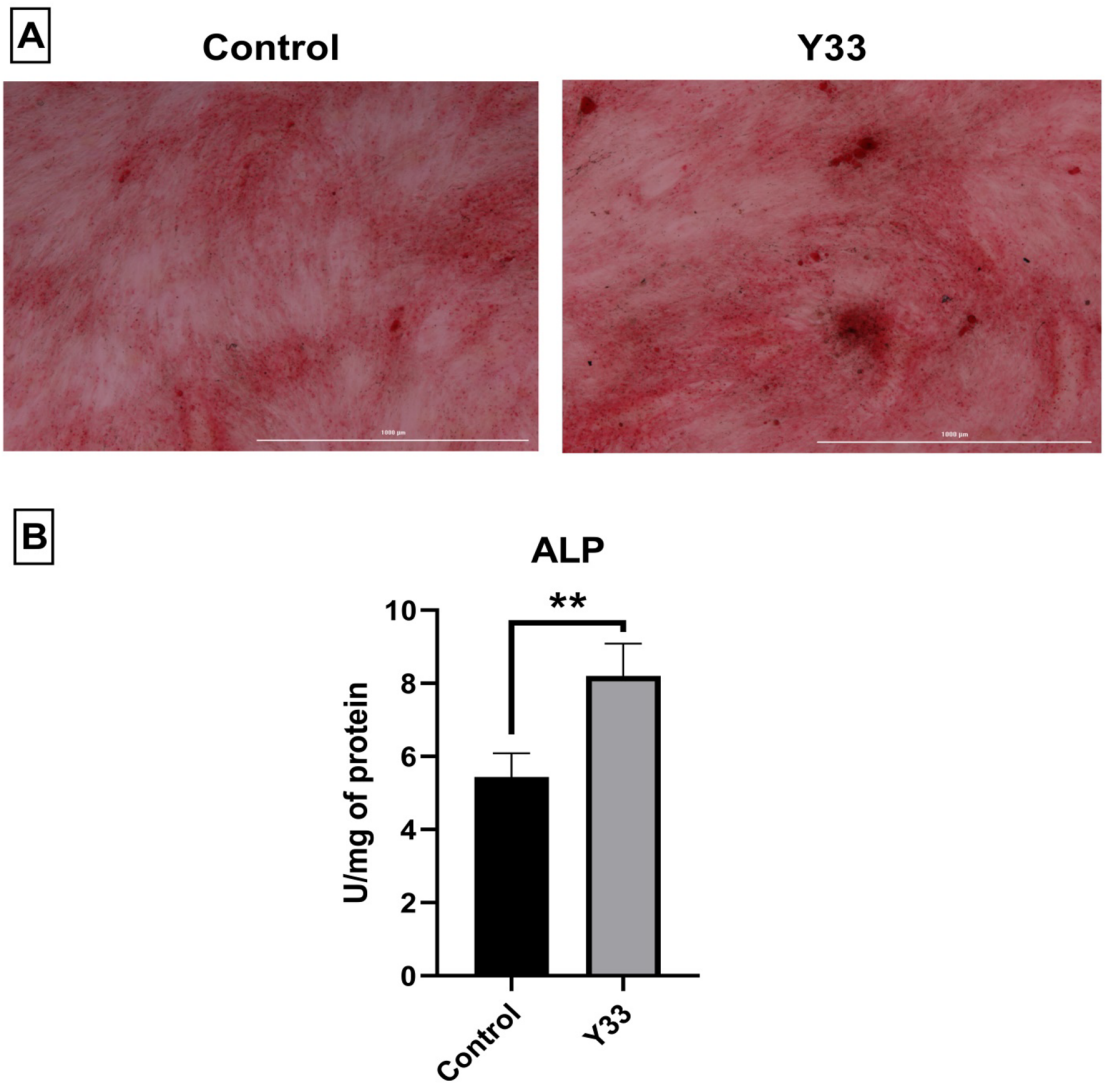
Alizarin Red staining (**A**) and alkaline phosphatase activity (**B**) of the treated groups by the extract. The white bars under microscopy images are equal to 1000 µm. **P < *0.01.*

Based on the Alizarin red staining results (Fig. 2A), the probiotic extract enhanced the osteogenic differentiation of ADSCs compared to the control group. As shown in Fig. 2B, the ALP activity in the probiotic-treated group was significantly higher than in the control group. In addition, the probiotic extract caused a significant increase in the expression levels of RUNX2 and osteocalcin and also caused a rise in the protein levels of collagen I and FGF-23 (osteocyte-specific markers) in comparison to the control group (Fig. 4A,C). All these results indicated that our probiotic extract increased the osteogenesis process, which is in line with previous studies. Tyagi et al. investigated the effect of *Lactobacillus rhamnosus* GG (LGG) supplementation on bone homeostasis in eugonadic young mice. Results indicated that LGG caused an increase in the volume of trabecular bone in mice because of enhanced bone formation.

It has also been shown that *L. rhamnosus* GG rescues the proliferation and osteogenic differentiation of mandible‐derived mesenchymal stem cells (MMSCs) and thereby protects against mandibular bone loss (induced by tenofovir) in mice. The Kim et al. study demonstrated that *Enterococcus faecium* L15 exopolysaccharide enhances the osteogenesis of human dental pulp stem cells through the p38 MAPK signaling pathway^76^.

In 2021, Yeom et al.^77^ reported that the surface proteins of *Propionibacterium freudenreichii* MJ2, which was isolated from raw milk, enhanced osteoblast differentiation and mineralization in the human fetal osteoblast cell line hFOB 1.19 through the BMP2/RUNX2 signaling pathway. Also, they showed that heat-killed MJ2 (hkMJ2) increases the bone mineral density of ovariectomized rats. Their results proposed the utility of hkMJ2 for improving bone health and treating osteoporosis. In the present study, the cell source is different from Yeom et al. study. We used human adipose-derived mesenchymal stem cells that have multi-lineage differentiation capacity.

Our results showed that the expression level of RUNX2 in the probiotic-treated group was significantly increased compared to the control group. This suggests that our new probiotic extract, similar to *P. freudenreichii* MJ2, increases osteogenic differentiation via the BMP2/RUNX2 signaling pathway.

Adipose tissue is known as a source of energy storage and one of the most important endocrine organs that releases many metabolic mediators. These metabolites include hormones, cytokines, chemokines, and other immune-related factors. As a result, hypertrophy (increased fat volume) and hyperplasia (increased number of fat cells) in the process of obesity cause overproduction of these compounds, which causes poor cellular messaging throughout the body. Fatty hyperplasia, which is strongly associated with obesity, is due to the fat differentiation of mesenchymal stem cells (MSCs)^78^. Therefore, in the process of obesity, both the number and size of fat cells increase, resulting in the differentiation of pre-adipocyte cells.

As previously mentioned, the expansion of marrow adipose tissue (MAT) and the related lipotoxicity are significant causes of bone loss. Various conditions, including diabetes, obesity, anorexia nervosa, glucocorticoid therapy, osteoporosis, and Cushing syndrome, can accelerate the accumulation of adipose tissue in bone marrow^79^. For this reason, in addition to osteogenesis, we also investigated the effect of this new probiotic extract on the adipogenesis process, to get a better understanding of its role in preventing obesity and, thus osteoporosis.

### Adipogenesis and the extract treatment

**Figure 3:**
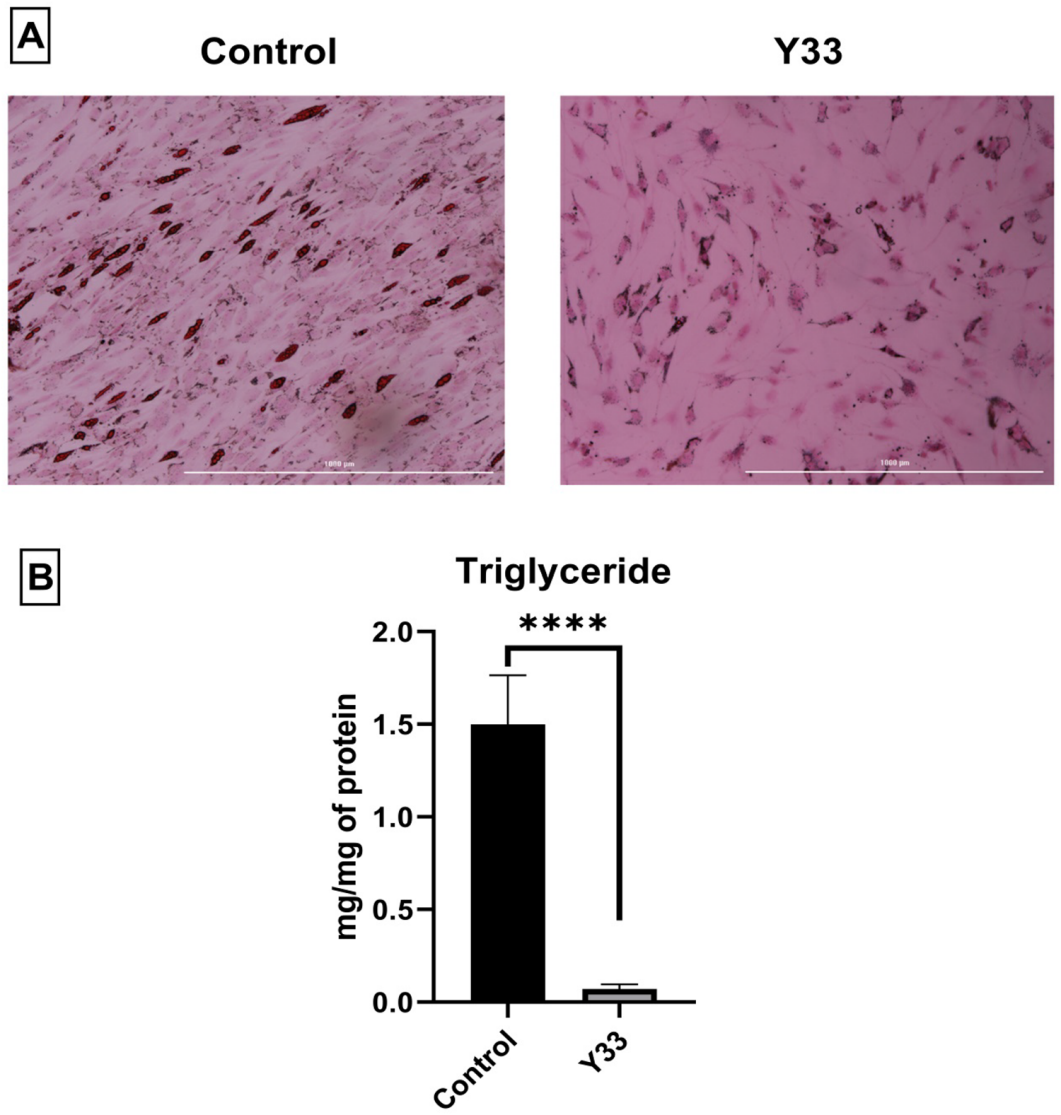
(**A**) Oil Red O staining and (**B**) triglycerides content of the treated groups by the extract. The white bars under microscopy images are equal to 1000 µm. ****P < *0.0001.*

**Figure 4:**
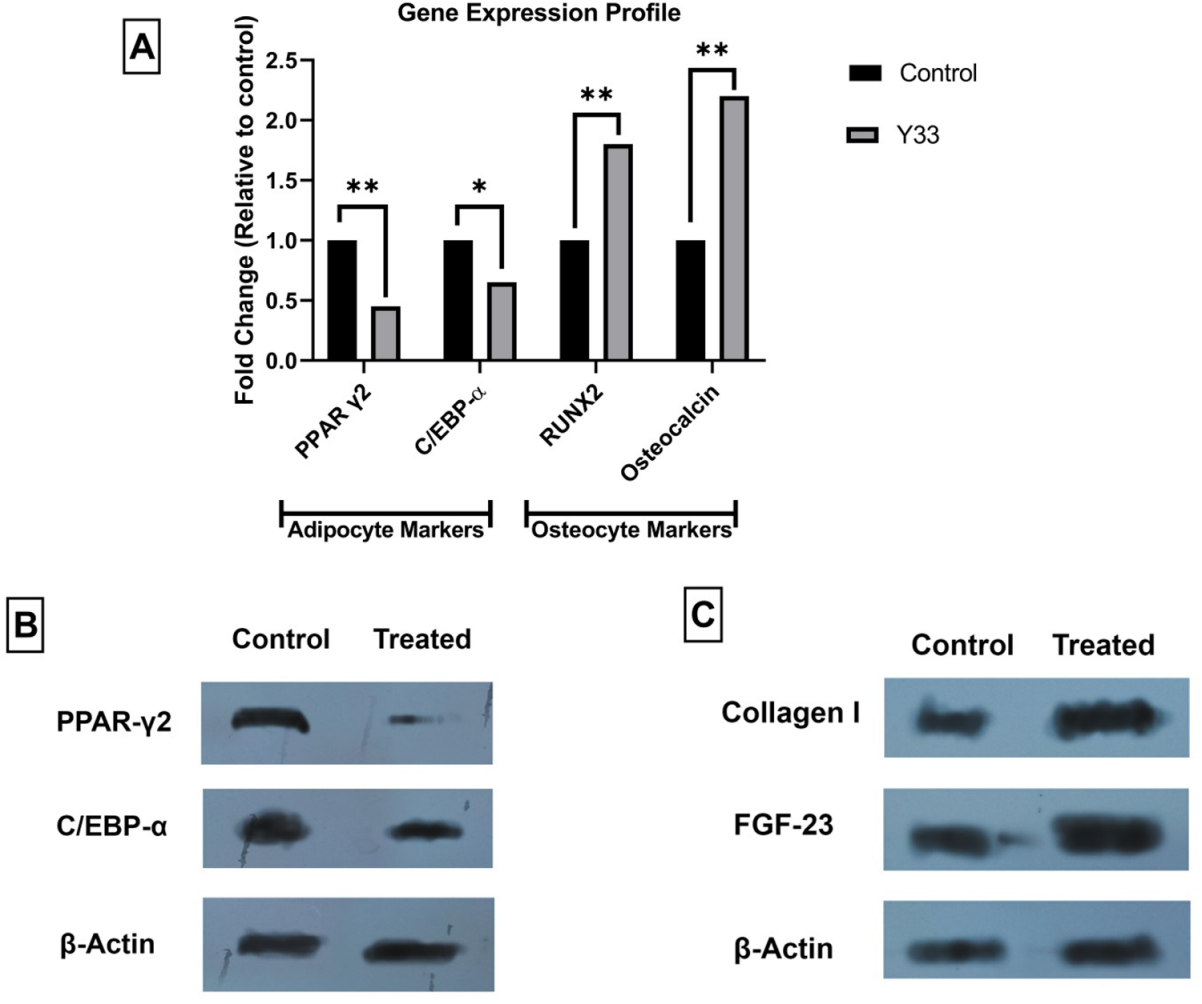
(**A**) qRT-PCR of adipogenic and osteogenic markers of the treated groups. Western blot assay of the adipogenic (**B**) and osteogenic (**C**) markers. The blots in the B and C parts of the figure are cropped to give a better comparative view. Original un-cropped images can be observed in Supplementary Fig. 1. *P < *0.05*, **P < *0.01.*

The results of oil red O staining indicated that the probiotic extract has inhibited lipid accumulation in ADSCs (Fig. 3A). As shown in Fig. 3B, the amount of triglyceride in the probiotic-treated group was significantly lower than in the control group. Also, the gene expression and protein levels of C/EBP-α and PPAR-γ2 (adipocyte-specific markers) were significantly decreased in the probiotic-treated group compared to the control group (Fig. 4A,B). According to these results, this new probiotic extract inhibits the adipogenesis process.

Our findings are consistent with prior observations that have demonstrated the inhibitory effects of probiotics on the adipogenesis process. For example, *L. plantarum* Q180 inhibits the adipogenesis of 3T3-L1 cells and decreases the adipocyte cell size, and as a result, it may have a positive influence on obesity^80^. Lee et al.^81^ demonstrated that *L. plantarum* L-14 (KTCT13497BP) extract significantly prevents the differentiation of mouse pre-adipocyte 3T3-L1 cells and human bone marrow mesenchymal stem cells (hBM-MSC) into mature adipocytes. Another study revealed that probiotic *E. faecalis* AG5 isolated from the rectal swab of Wistar rats reduces the obesity caused by a high-fat diet in Wistar rats. Also, the propionic acid produced by AG5 stimulates apoptosis in the 3T3-L1 pre-adipocytes^82^. It has also been shown that some species of *Bifidobacterium* or *Lactobacillus* produce normal weight (*B. animalis*), while others are associated with obesity (*Lactobacillus reuteri*)^83^. *B. animalis* subsp. *lactis* CECT 8145 has a strong fat reduction capacity that regulates fat metabolism in obese mouse models 9. *Lactobacillus acidophilus* NS1 has been able to improve obesity due to a high-fat diet in mice by improving fat metabolism and insulin sensitivity by the AMP-activated protein kinase pathway^84^.

Our new probiotic extract prevents osteoporosis by preventing obesity and the accumulation of adipose tissue in the bone marrow. We used a new probiotic strain that has not been isolated before, and no studies have been conducted to determine its potential properties so far. Unlike other probiotics studied, this one is isolated from dairy products. Also, we performed experiments on human adipose-derived mesenchymal stem cells that are capable of multi-lineage differentiation.

Previous studies examined the impact of their probiotic strain only on the osteogenesis or adipogenesis processes alone, while in this study we investigated the effect of our new probiotic extract on both processes. Therefore, we can achieve a better understanding of its properties in treating osteoporosis. Our findings suggest that this new probiotic extract can be used to prevent weight gain and obesity, as well as to prevent and treat osteoporosis.

## Conclusions

The findings showed that among the investigated LAB bacteria, *L. delbrueckii* subsp. *lactis* KUMS Y33 isolated from traditional fermented yogurt as a safe strain had the best probiotic score (*P* < *0.05*), including high tolerance to low pH and bile salt in digestive conditions (> 92%), favorable anti-pathogenic activity (All seven selected pathogen), good cell surface hydrophobicity (> 67%), high cell adhesion (> 48%), cholesterol absorption (> 63%), ideal auto- and co-aggregation (> 61%), and acceptable antibiotic susceptibility (All ten selected antibiotics). Also, secreted substances from this strain effectively inhibited the differentiation of human mesenchymal stem cells into adipocytes. Hence, this *Lactobacillus* strain has enough capacity and potential to be introduced and used as a probiotic. On the other hand, results proved that 16S-rRNA gene sequencing with high detection power can be used as an effective, low-cost, and rapid alternative for the identification and differentiation of *Lactobacillus* strains isolated from dairy products. The extract of *L. delbrueckii* subsp. *lactis* KUMS Y33 also promotes osteogenesis in parallel with adipogenesis inhibition comparing to control group (*P* < *0.05*).

According to the results of the present study, the probiotic extract inhibits adipogenesis and significantly increases osteogenesis, suggesting a positive role in the prevention and treatment of bone aging, including conditions such as osteoporosis. However, total implications in body will remain unclear as a complex system in body will limit the effects. It is also cannot be concluded which compound in the extract effect the trends. This extract can be provided as soft gel and be implicated for future in-vivo studies and fractionation will also provide more insight of the potential. Osteoporosis is a common condition characterized by a decrease in bone density and deterioration of bone tissue, which increases the likelihood of fractures. Recent research suggests that probiotics, particularly certain strains of *Lactobacillus* like the ones identified in our study, may offer significant advantages for maintaining bone health. Incorporating probiotics, especially specific *Lactobacillus* strains, into osteoporosis treatment strategies holds promise for further investigation. However, further research is required to fully understand the underlying mechanisms and confirm the validity of these findings.

### Supplementary Information

Supplementary Figure 1.

## Data Availability

The datasets generated and/or analysed during the current study are available in the NCBI GeneBank repository, accession numbers OQ826785 (https://www.ncbi.nlm.nih.gov/nuccore/OQ826785), OQ826808 (https://www.ncbi.nlm.nih.gov/nuccore/OQ826808), OQ826809 (https://www.ncbi.nlm.nih.gov/nuccore/OQ826809), OQ826810 (https://www.ncbi.nlm.nih.gov/nuccore/OQ826810), and OQ826827 (https://www.ncbi.nlm.nih.gov/nuccore/OQ826827).
