# Supplementary figures and images for: Potential probiotic Lactobacillus delbrueckii subsp. lactis KUMS-Y33 suppresses adipogenesis and promotes osteogenesis in human adipose-derived mesenchymal stem cell
Source: Sci Rep. 2024 Apr 27;14:9689. doi: 10.1038/s41598-024-60061-2 (PMC11055903; doi:10.1038/s41598-024-60061-2)

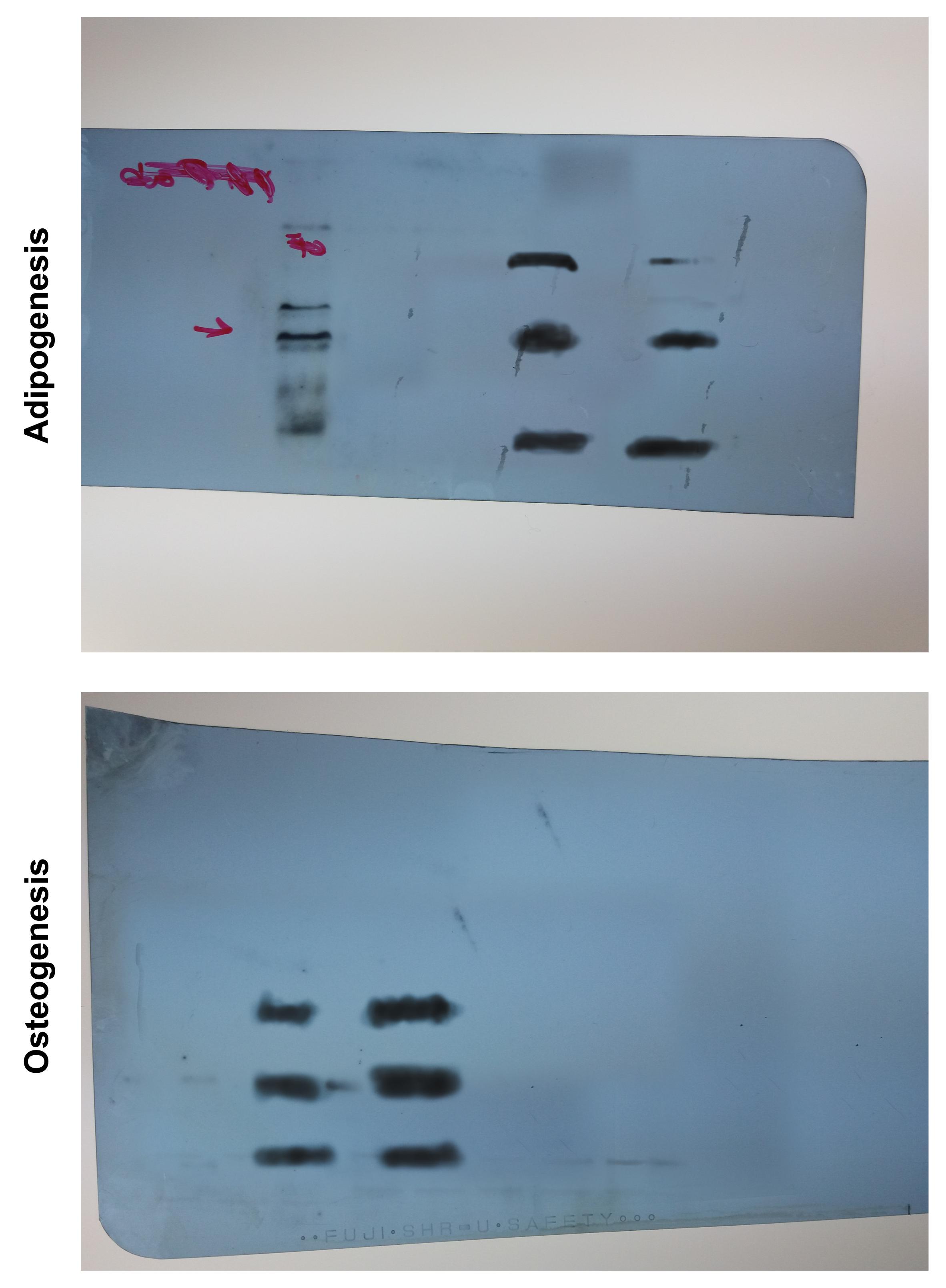

Supplement: Supplementary file 1 — Supplementary Figure 1. [file 41598_2024_60061_MOESM1_ESM.jpg]
